# The Neurophysiological Processing of Music in Children: A Systematic Review With Narrative Synthesis and Considerations for Clinical Practice in Music Therapy

**DOI:** 10.3389/fpsyg.2021.615209

**Published:** 2021-04-15

**Authors:** Janeen Bower, Wendy L. Magee, Cathy Catroppa, Felicity Anne Baker

**Affiliations:** ^1^Faculty of Fine Arts and Music, The University of Melbourne, Melbourne, VIC, Australia; ^2^Brain and Mind, Clinical Sciences, The Murdoch Children's Research Institute, Melbourne, VIC, Australia; ^3^Music Therapy Department, The Royal Children's Hospital Melbourne, Melbourne, VIC, Australia; ^4^Boyer College of Music and Dance, Temple University, Philadelphia, PA, United States; ^5^Melbourne School of Psychological Sciences and The Department of Paediatrics, The University of Melbourne, Melbourne, VIC, Australia; ^6^Psychology Department, The Royal Children's Hospital Melbourne, Melbourne, VIC, Australia; ^7^Centre of Research in Music and Health, Norwegian Academy of Music, Oslo, Norway

**Keywords:** systematic review, music, brain imaging, child, music therapy, acquired brain injury

## Abstract

**Introduction:** Evidence supporting the use of music interventions to maximize arousal and awareness in adults presenting with a disorder of consciousness continues to grow. However, the brain of a child is not simply a small adult brain, and therefore adult theories are not directly translatable to the pediatric population. The present study aims to synthesize brain imaging data about the neural processing of music in children aged 0-18 years, to form a theoretical basis for music interventions with children presenting with a disorder of consciousness following acquired brain injury.

**Methods:** We conducted a systematic review with narrative synthesis utilizing an adaptation of the methodology developed by Popay and colleagues. Following the development of the narrative that answered the central question “what does brain imaging data reveal about the receptive processing of music in children?”, discussion was centered around the clinical implications of music therapy with children following acquired brain injury.

**Results:** The narrative synthesis included 46 studies that utilized EEG, MEG, fMRI, and fNIRS scanning techniques in children aged 0-18 years. From birth, musical stimuli elicit distinct but immature electrical responses, with components of the auditory evoked response having longer latencies and variable amplitudes compared to their adult counterparts. Hemodynamic responses are observed throughout cortical and subcortical structures however cortical immaturity impacts musical processing and the localization of function in infants and young children. The processing of complex musical stimuli continues to mature into late adolescence.

**Conclusion:** While the ability to process fundamental musical elements is present from birth, infants and children process music more slowly and utilize different cortical areas compared to adults. Brain injury in childhood occurs in a period of rapid development and the ability to process music following brain injury will likely depend on pre-morbid musical processing. Further, a significant brain injury may disrupt the developmental trajectory of complex music processing. However, complex music processing may emerge earlier than comparative language processing, and occur throughout a more global circuitry.

## Introduction

In recent years the number of publications reporting the neural mechanisms involved in music processing has grown exponentially as scientists seek to systematically map the “implicit musical ability of the human brain” (Koelsch et al., 2000, p. 539; Koelsch, 2014, p. 170; Albusac-Jorge and Giménez-Rodríguez, 2015). The impact of music on a broad range of brain processes supports the therapeutic use of music in clinical neurologic populations; including neurorehabilitation, neuro-palliative rehabilitation, and neuro-psychiatry (Altenmüller and Schlaug, 2012; Thaut and Hoemberg, 2014; Chorna et al., 2019). However, current music neuroscience and clinical evidence pertains predominantly to the adult population.

A significant body of behavioral-based evidence indicates that fundamental auditory processing and musical communication are inherent in infants (Stewart, 2008; Trehub, 2010). It is widely accepted that the ability to meaningfully process music is acquired effortlessly from infancy without a requirement for formal training (Stewart, 2008). However the development of the neurophysiological correlates of music processing in childhood remains under-researched and poorly understood (Koelsch et al., 2003; Jeng et al., 2016a). Without a foundational knowledge of music processing in children it is not possible to understand the impact of an acquired brain injury (ABI) on the ability to process music and the potential for interruption to the ongoing development of musical processing. Additionally, a foundational knowledge is required to understand potential clinical implications of music interventions targeted to support recovery and rehabilitation following ABI.

Music is a complex auditory stimulus that consists of various components including melody, rhythm, timbre, and harmony (Altenmüller and Schlaug, 2013). When adults listen to music, these components are perceived as a phenomenological whole, however, they are processed separately through a complex bilateral network of cortical and subcortical regions (Altenmuller, 2001; Peretz and Zatorre, 2005; Levitin and Tirovolas, 2009). The neural substrates underpinning music perception and production vary depending on the musical task, as there is no single music center of the adult human brain (Tramo, 2001; Peretz and Zatorre, 2005; Altenmüller and Schlaug, 2013; Särkämö et al., 2013). This global processing supports the hypothesis that some ability to meaningfully perceive and process music will remain intact despite significant neural damage, potentially making music a unique therapeutic medium for individuals following ABI (Sihvonen et al., 2017).

A child's brain is not simply a smaller version of an adult's brain. Childhood is a period of rapid development and children's brains vary in shape and tissue composition compared to adult brains (Wilke et al., 2003). However, early plasticity theories are insufficient to describe the observed patterns of increased morbidity and cognitive impairment in young children following diffuse ABI (Crowe et al., 2012). Thus, the predominate theory of recovery following ABI in children supports that the developing brain of a child is at a significantly greater risk of adverse outcomes following severe ABI (Anderson and Yeates, 2010). Therefore, adult theories of recovery and rehabilitation cannot be directly translated to the pediatric population. This review was undertaken to develop a theoretical foundation for music-based interventions with children with a severe ABI, particularly those presenting with a disorder of consciousness (DOC).

## Objective

This systematic review with narrative synthesis was conducted to address the central question “what does brain imaging data reveal about the receptive processing of music in children?” Following the development of the narrative to answer this question, the implications of the results for children with severe ABI are explored. This review is a first step in developing a theory about the neural processing of music in children to support clinical practice in music therapy.

## Method

### Literature Searches

The search strategy was developed in consultation with a medical research librarian. Systematic searches of electronic databases were conducted to retrieve peer-reviewed references for inclusion in the first round of screening. Medline (Ovid), Embase (Ovid), PsycINFO (Ovid), Cochrane, and CINAHL (Ebsco) were searched predominantly using thesaurus terms to maximize the number of retrieved studies. Pubmed was searched, using keywords only, to retrieve items not indexed in Medline. The Medline search strategy was adapted for searching all other databases (see Appendix A for Medline Search Strategy). Thesaurus terms for searching Medline were categorized around four concepts:

- Brain
- Brain/neuro imaging techniques
- Music
- Child (0-18 years of age)

The four concepts were combined using the Boolean “AND.” Results were limited to the English language and children aged 0-18 years. Additional limiters for animals and participants with neuro-developmental disorders and hearing loss were applied. Additional items were identified through hand searching reference lists of relevant retrieved articles. Results from these searches were imported to bibliographic management software (Endnote X8, Thomson Reuters).

### Inclusion Criteria

Inclusion criteria were intentionally broad to maximize the data available for synthesis. References published in the last 20 years, reporting both electrical and hemodynamic brain imaging data, about the processing of music in neurologically healthy children, aged 0-18 years, and reported in peer-reviewed journals were included. References that reported evoked responses, neural structures, and networks and/or processes involved in the perception and processing of music and/or its individual elements were included. This included single or pure tones, as single tones are the foundation of melody. Further, the perception of music, as opposed to the production of music or active music making, was explored because music-based interventions supporting early recovery from an ABI are likely to involve passive listening rather than active music production (Rollnik and Altenmüller, 2014). See Appendix B for full inclusion and exclusion criteria, and Appendix C for the rationale for the inclusion/exclusion criteria.

### Study Selection

Two reviewers independently screened the titles and abstracts of all retrieved references, applying the inclusion and exclusion criteria to determine inclusions for second-round review. The reviewers recorded their decision in a pre-formatted Microsoft Excel document. These results were compared, and in the instance of disagreement a third reviewer independently screened the reference. Once a consensus about reference inclusion was reached, the full-text articles were retrieved for a second round of screening. Again, two reviewers independently screened all retrieved full-text articles with a third reviewer sought for any discrepancies, to determine the full-text inclusions for data extraction and synthesis. Data were extracted from the full-text references into a pre-formatted Microsoft Excel document by the first author.

### Data Synthesis

The development of the narrative synthesis was based on an adaptation of the “Guidance on the Conduct of Narrative Synthesis in Systematic Reviews” developed by Popay et al. (2006). This model supports a text-based approach to the process of data synthesis, and was suited to synthesizing data extracted from studies that report a diverse range of evidence (Popay et al., 2006). In this instance this included diverse imaging methods, diverse musical stimuli, and a broad age range. The Popay et al. (2006) guidance is best suited to synthesis of intervention studies, and given the broad intention of this review, the review was not limited to intervention studies. Therefore, only the second (developing a preliminary synthesis) and third (exploring relationships in the data) steps of the guidance were undertaken.

## Results

**Figure 1:**
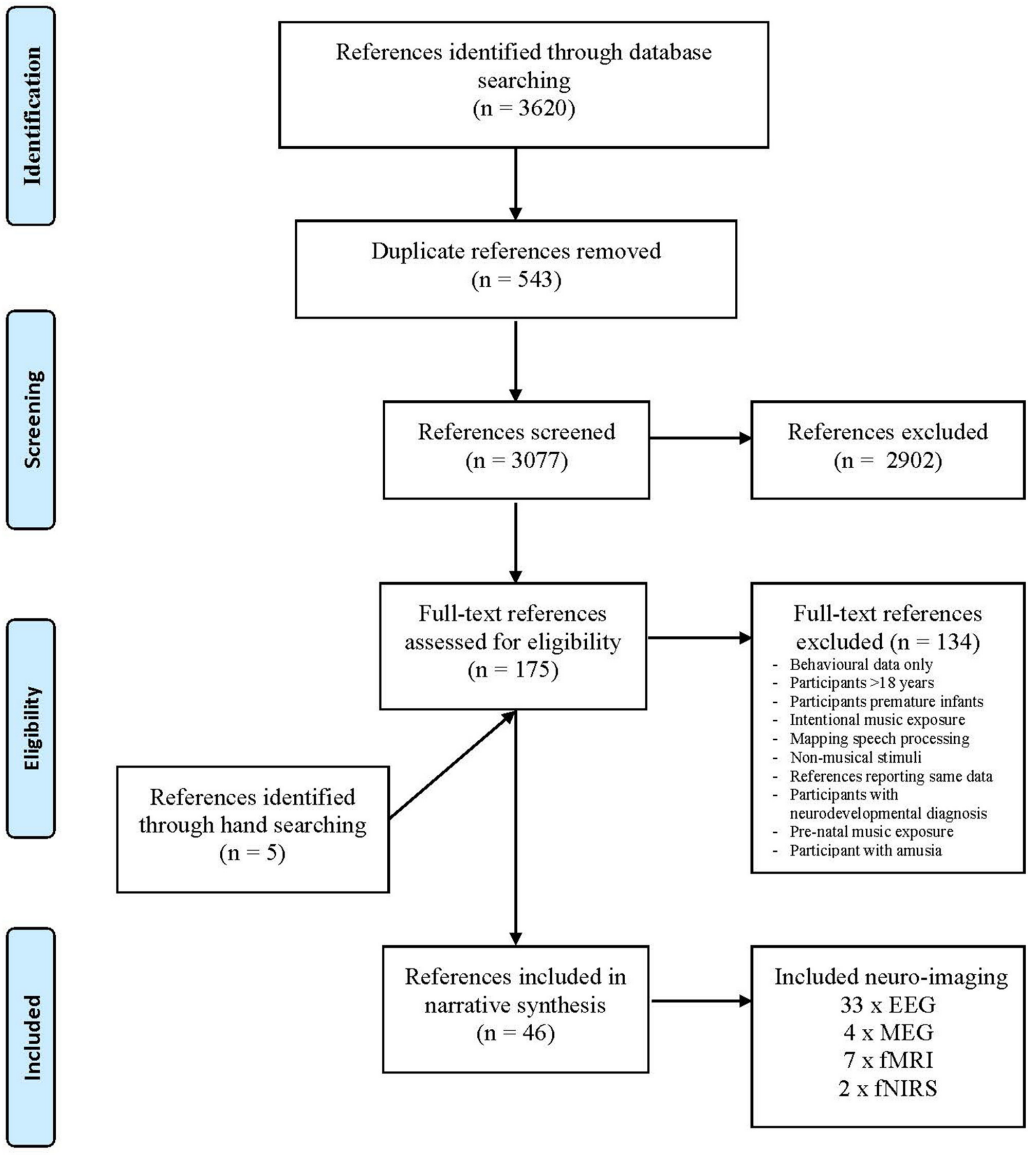
Reference selection process. Adapted from Moher et al. (2009).

Database searches were conducted on May 3, 2019, and updated on June 21, 2020. A total of 3,620 references were retrieved, 543 duplicates were removed, resulting in an initial library of 3,077 references. These references were screened and 175 references were included in the second-round full-text screen. After the full-text screen, 41 references were included in the synthesis. An additional five references were located through hand searching. The synthesis included 46 references that employed the following imaging techniques (see Figure 1):

- 33 references employed EEG scanning
- 4 MEG
- 7 fMRI
- 2 fNIRS^1^

All participants in the included references were healthy neuro-typically developing children aged 0–18 years and were not reported to have received formal musical tuition. In references where useful data about the healthy control group were presented as a comparison to a clinical population, data about the healthy controls were included. In references where the intervention group received musical training or intentional musical exposure, but there was useful data reported about the control group who did not receive musical training, data from the control group were included. In the instance where references reported the same data, the later reference was excluded, for example Overy et al. (2004, 2005).

### Narrative Synthesis

The included references were grouped into age categorizations approximating Piaget's stages of cognitive development (see Table 1):

- Infants aged 0–24 months (24 references; 3 fMRI, 2 NIRS, 1 MEG, and 18 EEG)
- Young children aged 2–6 years (8 references; 3 fMRI, 1 MEG, and 4 EEG).
- Children aged 7–12 years (11 references; 1 fMRI, 2 MEG, and 8 EEG)
- Adolescents aged 13+ years (3 references; 3 EEG)

**Table 1:** Included references summary of results (ordered by year of publication).

**References**	***n***	**Age**	**Scanning methodology**	**Music stimuli**	**Summary of primary neurophysiologic response/s**
**INFANTS AGED 0-24 MONTHS–24 REFERENCES INCLUDED**					
**fMRI−3 references included**					
Anderson et al. (2001)	14	7d^a^ Mean	1.5T; 5 mm slices Voxel size not provided Natural non-sedated sleep ROI^b^: Superior temporal region	Frequency modulated tones	BOLD response detected in ROI in *n* = 14 - 9/14 BOLD signal increase - 5/14 BOLD signal decrease
Perani et al. (2010)	18	3d Mean	1.5T; 23 slices 3.75 × 3.75 × 3 mm voxel Natural non-sedated sleep	Western Classical Music • Matched counterparts that varied in degree of consonance/dissonance	- Hemispheric asymmetry present R>L for original music - Peak activation in R primary auditory cortex extending to secondary auditory cortex - R auditory cortex and R amygdala-hippocampal activation stronger to original music compared to altered. L limbic structure activation greater to dissonant music
Dehaene-Lambertz et al. (2010)	7	72d Mean	1.5T; 16 slices 4 mm isotropic voxel Settled awake and natural sleep ROI: planum temporale	Mozart piano sonatas • Maternal and unfamiliar voice (music as comparative stimuli)	- Bilateral activations in ROI - Music stimulated greater R activation than speech conditions
**fNIRS−2 references included**					
Kotilahti et al. (2010)	13	1.8d Mean	16 channel Natural non-sedated sleep	Mozart piano concerto • Female infant directed speech (music as comparative stimuli)	- 11/13 significant hemodynamic response to stimuli - No significant lateralisation of hemodynamic response to either music or speech - 3/13 music elicited a strong hemodynamic response - Mixed positive and negative hemodynamic responses
Homae et al. (2012)	46	3&6m	Multi channel Natural non-sedated sleep ROI: R temporoparietal region	Digitally recorded chromatic tone sequences	- Bilateral temporal activation to stimuli - 3 month olds detected pitch changes in successive tones - ROI had increased sensitivity to auditory sequences with age
**MEG−1 reference included**					
Muenssinger et al. (2013)	15	34d Mean	156 Sensors Natural sleep or settled awake Stimuli presented monaurally to L	500 Hz tones 750 Hz disinhibitor tone	- Nil evidence of auditory habituation - MMN^1^ elicited in response to infrequent 750 Hz tone
**EEG−18 references included**					
EEG/Auditory Evoked Potential (AEP)					
Schmidt et al. (2003)	167	3-12 m Range	Auditory evoked potential (AEP)^2^ Recorded at 4 electrodes Awake protocol	Orchestral music–sad, fear & joy: 1. Adagio, Barber 2. Peter and the Wolf, Prokofiev 3. Spring, Vivaldi	- AEP not altered by different emotional valance of stimuli - 3 month olds increased activation, 12 month olds deactivation in response to stimuli - More frontal than parietal activation at 9 & 12 months - No significant lateralisation effect
Jing and Benasich (2006)	19	3-24 m Range	Auditory evoked potential Recorded at 62 electrodes Awake passive protocol	Oddball paradigm–pitch deviants • Standard−100 Hz complex tones • Deviant−300 Hz	- Significant morphological changes of AEP at 6-7 months - P150, N250, P350, N450 reliably elicited after 6 months - MMN not consistent at 3 months - MMN robust and more adult like after 6 months - MMN more prominent R frontal than L - Latency of AEP components decreased with age
Cirelli et al. (2016)	60	7 m Mean	Auditory evoked potential Recorded at 124 electrodes Awake passive protocol	Six beat rhythmic pattern of ambiguous meter	- Entrainment to rhythm - Perception of rhythm in duple OR triple meter
EEG/AEP/Mismatch Negativity (MMN)					
Cheour et al. (2002)	10	2-6dRange	AEP**/**Mismatch negativity (MMN) Recorded at 13 electrodes Natural non-sedated sleep	Oddball paradigm–duration deviants • Standard−100 ms • Short deviants−40 ms • Long Deviants−200 ms	- Standards elicited P200 peaking 300–350 ms - MMN elicited in all infants to both long and short deviants - MMN long latency
He et al. (2007)	39	2,3,4m	AEP**/**MMN Recorded at 124 electrodes Awake passive protocol	Oddball paradigm—pitch deviants • Synthesized piano tones • Standard–C5 (523 Hz) • Deviant–F#5 (740 Hz)	- At 2 months slow positive wave elicited by deviants - At 3 & 4 months MMN-like negativity elicited by deviant - MMN-like negativity followed by P3a - MMN-like wave stronger in R hemisphere - MMN-like wave amplitude increased and latency decreased with age
He et al. (2009a)	29	2&4m	AEP**/**MMN Recorded at 124 electrodes Awake passive protocol	Oddball paradigm—interval deviants • Synthesized piano tones • Standard–ascending interval C5-F#5 • Deviant–descending F#5–C5	- At 2 months no significant MMN, standard and deviant waves not significantly different - At 4 months MMN-like negativity in response to deviants - MMN-like response larger peak amplitude than adults - MMN-like response longer latency than adults - Trend for R hemisphere dominance of MMN-like response
He et al. (2009b)	67	2&4m	AEP**/**MMN Recorded at 124 electrodes Awake passive protocol	Oddball paradigm—pitch deviants • Synthesized piano tones • Standard—C5 (523 Hz) • Deviant—F#5 (740 Hz) • Fast and slow presentations	- At 2 months no significant difference in response peaks between standard and deviant pairs - At 4 months MMN to deviants had similar function characteristics to adult MMN - Presentation rate of stimuli had no impact on MMN amplitude but MMN had longer latency to fast presentation rate (4 month olds)
He and Trainor (2009)	59	3,4,7m	AEP**/**MMN Recorded at 124 electrodes Asleep /Awake passive protocol	Oddball paradigm –pitch deviants • Standard—Ascending tone pairs with fundamental frequency present • Deviants—Tone pairs with missing fundamental frequency	- *If missing fundamental frequency of deviant perceived MMN elicited as pitch would descend (standard has ascending pitch)* - At 3 months—no MMN to deviants - At 4 & 7 months MMN elicited to deviants, therefore missing fundamental perceived
Stefanics et al. (2009)	10	2-3d Range	AEP**/**Discriminatory response Recorded at 3 electrodes Natural non-sedated sleep	Oddball paradigm—interval deviants • Standard—descending pitch 2 semitones • Deviants—descending pitch 7 semitones	- AEP elicited by deviants significantly different to those evoked by standard intervals (precursor to MMN)
Tew et al. (2009)	17	6.3m Mean	AEP**/**Mismatch negativity (MMN) Recorded at 128 electrodes Awake passive protocol	Oddball paradigm—melody deviants • Standard—4 note melody transposed to 20 starting notes • Deviants—final note raised by semitone	- Extended right frontally positive response to deviants - Immature MMN-like response
Winkler et al. (2009)	24	2-3d Range	AEP**/**Mismatch negativity (MMN) Recorded at 3 electrodes Natural non-sedated sleep	Oddball paradigm—beat deviant • Standard—2 bars of 8 isochronous beats • Deviant—down beat omitted	- Deviant had syncopated feel - Deviant elicited discriminative waveform; negative peaks at 200 and 316 ms followed by positive at 428 ms - Immature MMN-like response
Hamalainen et al. (2011)	39	6m	AEP**/**Mismatch negativity (MMN) Recorded at 62 electrodes Awake passive protocol	Oddball paradigm—pitch deviants • Standard–tone pair, identical 100 Hz tones Deviant—tone pair 100/300 Hz • Deviant—down beat omitted	- Bilateral activation close to auditory cortex - Deviant stimuli elicited large positive-negative complex at 408 and 540 ms after tone pair
Virtala et al. (2013)	19	1.7d Mean	AEP**/**Mismatch response (MMR) Recorded at 11 electrodes Natural non-sedated sleep	Oddball paradigm—chord deviants • Standards—root position major triads • Deviants: 1. Root position minor triads 2. 2nd inversion major triad 3. Dissonant chords	- Statistically significant MMR to major/minor and dissonant /consonant chords - Dissonant deviants elicited frontal positive MMR - Minor deviants elicited negative MMR most pronounced in parietal regions - No MMR to major inversion deviants
Marie and Trainor (2013)	16	7.7m Mean	AEP**/**MMN Recorded at 124 electrodes Awake passive protocol	Oddball paradigm—pitch deviants • Standards—simultaneous tone pairs minor 10th apart • Deviants—one tone in standard pair shifted up or down 1 semitone • Also single voice condition	- MMN response elicited by deviants - MMN latency significantly longer for pair stimulus than single voice - MMN elicited when deviants presented in either voice of pair - MMN larger when deviant presented in high voice of pair compared to low voice
Marie and Trainor (2014)	16	3.5m Mean	AEP**/**MMN Recorded at 124 electrodes Awake passive protocol	Oddball paradigm—pitch deviants As per Marie and Trainor (2013) above	- Larger MMN recorded for high voice deviants than low voice deviants - Shorter MMN latency for deviants in high voice - High voice superiority
Háden et al. (2015)	33	1-3d Range	AEP**/**Mismatch response (MMR)^3^ Recorded at 9 electrodes Natural sleep or settled awake	Oddball paradigm—melody deviants • Standard—Trains 6 descending pitches • Deviants—Position 2 OR 5 pitch ascending	- Significant slow wave positive discriminative AEP elicited by deviants in Position 5 (discriminative MMR) - Nil significant response to deviants at Position 2 - Emerging predictive pitch processing
EEG/AEP/Frequency Following Response (FFR)					
Jeng et al. (2016a)	20	1-3d Range	AEP**/**FFR^4^ Recorded at 3 electrodes Natural non-sedated sleep	4 contrasting mono-syllabic tones with different pitch contours produced by male voice	- 20/20 infants displayed electrical response that followed the contours o the four stimuli/pitches
Jeng et al. (2016b)	13	1-3d & 3m	AEP**/**FFR Recorded at 3 electrodes Natural non-sedated sleep	Stimuli as per Jeng et al. (2016a) above	- 13/13 infants displayed clear spectral energy that followed pitch contour of 4 stimuli - 11/13 infants displayed improved tracking accuracy and pitch strength at 3 months of age
**CHILDREN AGED 2-6 YEARS**—**8 REFERENCES INCLUDED**					
**fMRI**—**3 references included**					
Overy et al. (2004)	33	6.33y Mean	3T; 26 slices 3.8 × 3.8 × 4 mm voxel Active**/**behavioral response	Marimba like tone • Melody stimulus—short musical phrase of five different pitches • Rhythm stimulus– single pitch with various durations to form rhythmic pattern	- Bilateral activation in superior temporal gyrus (auditory cortex) for both conditions - Hemispheric activations not significantly different for rhythm or melody - Designated ROI analysis—small region in R superior temporal gyrus significantly higher activation for melody than rhythm
Guerrero Arenas et al. (2016)	15	5-6y Range	1.5T; 35 slices Voxel size not reported Settled awake	Specifically composed consonant and dissonant piano melodies	- Both consonant and dissonant music activated temporal lobes, frontal lobes, fusiform and cerebellum - Larger activation in cerebellum and fusiform for consonant/tonal music - Consonant/tonal music stimulated induced larger activation in frontal lobes and limbic system - Dissonant/atonal music induced larger activation in temporal areas and cerebellum
Prabhakar et al. (2018)	22	28.9m Mean	3t: 46 slices 3 mm isotorpic voxel Analysis confined to hippocampus Natural non-sedated sleep	Unfamiliar African lullaby	- Presentation of previously presented lullaby stimulated bilateral hippocampal activation compared to novel song - No laterality effect observed
**MEG**—**1 reference included**					
Fujioka et al. (2006)	4	4–6y Range	151 sensors Settled awake Longitudinal design (scanned 4 times in a year)	440 Hz violin tone	- Longer latency auditory evoked field (AEF)^5^ for violin tone compared to noise burst - Violin tone—larger P100m and P320M peak amplitude compared to noise burst - Shorter peak latencies across sessions (3/4 monthly) for N250m, P320m and N450m - Decreased amplitude for N250m and p320m across sessions
**EEG−4 references included**					
Jentschke et al. (2008)	20	5.25y Mean	AEP**/**Early right anterior negativity (ERAN)^6^ Recorded at 22 electrodes Awake passive protocol	Midi produced piano, chord progressions with either: • Regular V-I/perfect cadence • Irregular II-I Cadence	- ERAN elicited in response to irregular supertonic cadence - ERAN prominent over frontal leads - ERAN followed by N5 also over frontal leads - ERAN longer latency than adults
Putkinen et al. (2013)	25	2.79y Mean	AEP**/**MMN Recorded at 5 electrodes Awake passive protocol (Overy et al., 2004)	Oddball paradigm—multiple deviant paradigm • Standard—500 Hz, 200 ms duration • Deviants—different frequency/.pitch, duration, gap, intensity, direction • Deviants—novelty sounds	- Temporal cues (duration and gap deviants) elicited significant MMN-like response followed by P3a-like and LDN-like response - No MMN-like response observed to frequency, intensity or direction deviants (pitch/melody based deviants) - Larger P3a response positively correlated with greater musical exposure
Jentschke et al. (2014)	62	30m Mean	AEP**/**ERAN Recorded at 21 electrodes Awake passive protocol	Midi produced piano, chord progressions with either: • Regular cadence V-I • Irregular cadence V-I • Irregular cadence V-ii6 (Neapolitan)	- ERAN peaking ~300 ms elicited for both irregular chords - ERAN more broadly distributed than reported in adults - ERAN had smaller amplitudes and longer latency than adults - No N5 observed following ERAN
Corrigall and Trainor (2014)	46	4.5y Mean	AEP**/**ERAN Recorded at 128 electrodes Awake passive protocol	Chord sequences & single line melodies • Standards follow Western Syntax • Atonal • Unexpected Key	- An early component (immature ERAN) elicited by syntactic irregularities in chord sequences but not melodies
**CHILDREN AGED 7**–**12 YEARS−11 REFERENCES INCLUDED**					
**fMRI−1 reference included**					
Koelsch et al. (2005)	10	10.2y Mean	3T; 24 slices 1.00. × 1.00 × 1.5 mm voxel Active**/**behavioral response	Midi produced piano tones Harmonic progressions: • Regular cadence (V7-1) OR • Irregular cadence (V7–ii6)	- Pattern of R hemisphere activation similar to non-musician adults - Recognition of musical syntax involves network comprising; pars opercularis in frontal lobes and interior ventrolateral pre-motor cortex in frontal lobes - 4 different cortical networks activated in music processing: 1. Musical structure inferior frontolateral cortex, superior temporal gyrus and pre-motor cortex 2. Musical meaning: posterior temporal areas 3. Working memory: supramarginal gyrus and pre-frontal cortex 4. Emotional aspects music: orbitofrontolateral cortex and anterior insula
**MEG−2 references included**					
Muenssinger et al. (2013)	22	9.7 y Mean	275 sensors Settled awake	Trains of 8 tones with single dishabituator tone at tone 6th position	- No habituation response observed - No decrease in signal between tones 1–5 - No signal increase from tones 5–7
Parviainen et al. (2019)	19	7–8 y Range	306 sensors Settled awake	Single 1,000 Hz tones presented at various inter-stimulus-intervals	- Sounds from contralateral ear elicit stronger response than ipsilateral ear - R hemisphere stronger and more adult-like AEF - Stronger activation emerges after 200 ms (slower than recorded in adults)
**EEG−8 references included**					
Ceponiene et al. (2002)	16	4 and 9 y	AEP Recorded at 62 electrodes Awake passive protocol	Harmonic tones • Short stimulus onset asynchrony (SOA) 700 ms • Long SAO 2.8-7.7 s	- AEP to short SOA at both ages consisted of P1 and N2 peaks - N2 amplitude decreased between 4 and 9 years - AEP to long SOA at 9 years consisted of P1, N1, P2, N2 peaks - N1 generators at 9 years anterior to that of adults - Amplitude and latency of P1 change as a function of age
Koelsch et al. (2003)	28	5 and9 y	AEP/ERAN Recorded at 12 electrodes Active/behavioral response	Chord progressions of 5 chords either: • Syntactically regular • ii6 at 3rd OR 5th position • Deviant instrumental sounds	- In by 5 and 9 year olds ii6 chord in 5th position elicited early anterior negativity (immature ERAN) - This ERAN –like response had *L* predominance in males and bilateral distribution in females - No ERAN to ii6 chord in 3rd position - Electrical correlates of music syntactic processing observed earlier than language equivalent
Magne et al. (2006)	10	8 y	AEP/ERAN Recorded at 28 electrodes Active/behavioral response	Children's repertoire and specifically composed melodies • Congruous ending • Incongruous ending (1/5 tone) • Incongruous ending (1/2 tone)	- No significant response to weakly incongruent endings (1/5 tone) - Strong incongruent ending (1/2 tone) elicited early negative component (immature ERAN) that was larger over R hemisphere
Jentschke and Koelsch (2009)	20	11.1y Mean	AEP/Early right anterior negativity (ERAN)^i^ Recorded at 22 electrodes Awake passive protocol	As per Jentschke et al. (2008)	- ERAN followed by N5 elicited by syntactically irregular chords - ERAN has increased latency and more bilateral scalp distribution compared to adults
Fox et al. (2010)	28	7–9y Range	AEP Recorded at 33 electrodes Awake passive protocol	• Single tone stimulus 1,000 Hz • Paired (identical) tone stimuli with increasing inter-stimulus intervals	- AEP in participants dominated by P1, maximal at front-central sites - Distinct response not observed when inter-stimulus interval <200 ms
Cirelli et al. (2014)	16	7.6y Mean	AEP Recorded at 128 electrodes Awake passive protocol	• Pure tones presented at 3 tempi • Isochronous presentation	- Patterns of desynchronization and rebound observed to two slower tempi - This pattern occurs most strongly in beta waves - Pattern of beta fluctuations followed tempo of stimulus (excluding fastest presentation) - Induced beta fluctuations entrained to isochronous auditory stimuli at slower tempi
James et al. (2015)	15	10.9y Mean	AEP/ERAN Recorded at 64 electrodes Active/behavioral response	Specifically composed string quartets • Regular ending (I) • Transgressed ending (inverted IV)	- No ERAN observed to transgressed endings - Centro-posterior negativity observed to transgressed cadences (similar to the semantic mismatch N400 - Strong activation in pre-motor areas for transgressed endings - Greater involvement L hemisphere than in adults
Alipour et al. (2019)	18	11.6y Mean	AEP Recorded at 32 electrodes Awake passive protocol	Monophonic instrumental melodies • Natural pieces (Hayden & Albinoni) • Same pieces with altered rhythmic patterns	- Synchronous activity between electrodes = functional connectivity - Dominant involvement of front-central and pre-motor areas - Alterations of functional connectivity due to rhythmic pattern change around front-central and inferior parietal regions
**CHILDREN AGED 13+** **YEARS−3 REFERENCES INCLUDED**					
**EEG−3 references included**					
Shahin et al. (2010)	46	4-25y Range	AEP Recorded at 20 electrodes Awake passive protocol	A3/220Hz & C3/131Hz: • Pure tones • Synthesized piano tones • Synthesized violin tones	- P1 latency decreased with age - N1 latency decreased with age - Phase locking for theta, alpha, beta and gamma bands strengthened with age - Phase locking at bands <20 Hz present at every age - Phase locking for bands >25 Hz present after 10 years
Mahajan and McArthur (2011)	87	10-17y Range	AEP Recorded at 30 electrodes Awake passive protocol	• 1,000 Hz pure tones • 1,200 Hz deviants	- Audible movie soundtrack had degrading effect on AEP of children and adolescents - Reliability of P2 amplitude and latency low in children and young adolescents - Reliability of N2 amplitude lower in children - N1 and P1 peaks amplitudes least affected by audible soundtrack
Yamazaki et al. (2018)	31	5&15y	AEP Recorded at 62 electrodes Awake passive protocol Monaural presentation	• Tone bursts (single tones) 500 Hz • Click trains	- At 5 years tones evoked immature P1 and N2 - At 15 years tones evoked P1, N1, P2, N2 peaks (similar to adult AEP) - At 5 years clicks elicited contralateral response and tones a bilateral response - At 15 years tones elicited R dominant processing and clicks continued to elicit contralateral response

1 *See Supplementary Material for description of the MMN component of the AEP/AEF*.

2 *See Supplementary Material for description of the AEP*.

3 *See Supplementary Material for description of the MMR component of the AEP*.

4 *See Supplementary Material for description of the FFR component of the AEP*.

5 *See Supplementary Material for description of the AEF*.

6 *See Supplementary Material for description of the ERAN component of the AEP*.

7 *See Supplementary Material for description of the ERAN component of the AEP*.

a *Age abbreviations: d, days of age/postnatal days; m, months of age; y, years of age*.

b *ROI, Region of interest*.

Where the study design was longitudinal or cross-sectional, the reference was included in the age category that includes the oldest age point in the study.

Results from the included references were initially explored in descriptive format by the first author. The narrative synthesis and discussion were then developed collaboratively with all authors, three of whom are experienced music therapists. Thus, the narrative gives consideration to data that are likely to be most pertinent to music therapists.

## The Neurophysiological Processing of Music in Children

**Figure 2:**
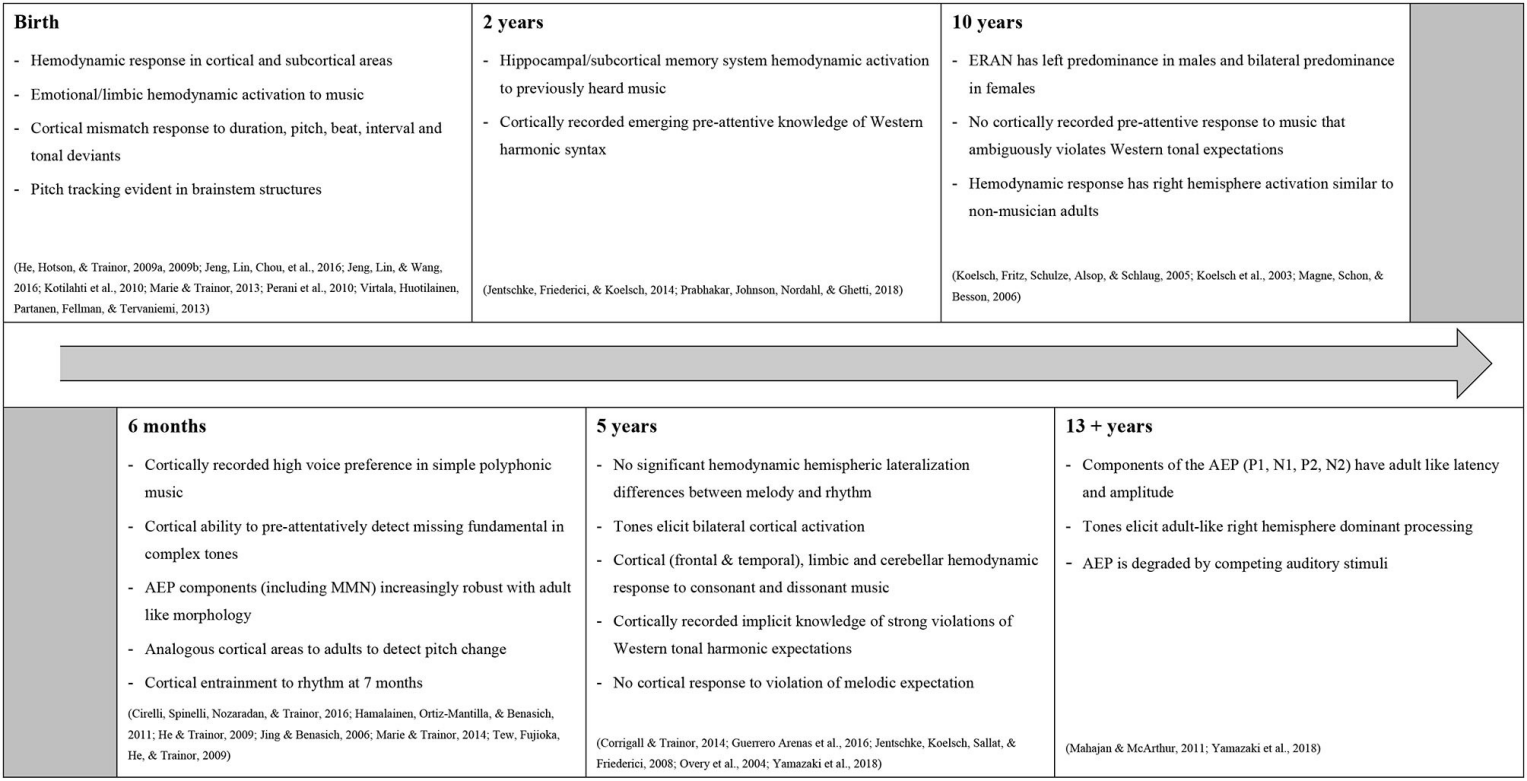
Timeline of significant developments of musical processing.

This narrative tracks musical processing across a timeline from full-term birth to adolescence, with the key musical milestones most relevant to clinical music therapy practice summarized below in Figure 2.

### Infants Aged 0–24 Months

Hemodynamic data obtained from fMRI and fNIRS scanning established that the infant brain processes music throughout a bilateral network of cortical and subcortical structures (Dehaene-Lambertz et al., 2010; Perani et al., 2010; Homae et al., 2012). Despite hemodynamic responses being mixed (negative/positive) because of immature vasculature, excerpts of classical music elicited a predominantly right hemispheric activation. Peak activation in the right auditory cortex extending into the secondary auditory cortex was reported, which is similar to patterns of temporal activation observed in adult non-musicians (Anderson et al., 2001; Kotilahti et al., 2010; Perani et al., 2010). Activation in the right insula and amygdala-hippocamal complex in the subcortical limbic system was also reported. This suggests music elicits an emotional response in newborns, albeit at a sensory level (Perani et al., 2010). Increasing sensitivity of the right temporoparietal region between 3- and 6-months to chromatic tone sequences may indicate ongoing development of a lateralization effect (Homae et al., 2012).

Distinct but immature electrical responses were recorded by EEG and MEG in infants, and a significant developmental milestone of these musically evoked cortical responses occurred around 6 months of age (Cheour et al., 2002; Jing and Benasich, 2006; Stefanics et al., 2009; Hamalainen et al., 2011; Muenssinger et al., 2013). The components of the auditory-evoked potential (AEP) in infants have longer latencies and greater amplitudes compared to those of older children and adults (He et al., 2009a,b; Marie and Trainor, 2013). However, the presence of a discriminatory response, or immature mismatch negativity (MMN), from 2 days of age to duration, beat, pitch, and interval deviants suggests a fundamental capacity for auditory working memory, pitch discrimination, and melody and beat perception (Cheour et al., 2002; Stefanics et al., 2009; Winkler et al., 2009; Muenssinger et al., 2013; Háden et al., 2015). Virtala et al. (2013) further reported that newborn infants display sensitivity to major-minor and consonant-dissonant tonalities. Mismatch responses with different cortical localizations were elicited by minor and dissonant deviants (Virtala et al., 2013). While noted that these mismatch responses likely indicate automatic discrimination and not preference, they nonetheless indicate cortical sensitivity to different tonalities (Virtala et al., 2013).

The cortical change mechanism for pitch discrimination is hypothesized to emerge around 3-4 months of age (He et al., 2009a,b). Prior to this, the discriminative slow wave positive response to single tone stimuli is likely a result of different neural generators (He et al., 2007). An adult-like MMN to single tone stimuli was recorded after 3 months and had a right hemisphere dominance, with the peak amplitude increasing and the latency decreasing as a function of age (He et al., 2007). Further maturation of the discriminatory response to single tone stimuli was reported at 4 months, with an MMN response elicited that was functionally similar to the MMN of adult controls (He et al., 2009b). The MMN elicited by more complex musical stimuli was reported to have a more protracted developmental trajectory, and at 4 months an intervallic stimulus elicited an immature MMN with a longer latency and increased amplitude compared to its adult counterpart (He et al., 2009a; Stefanics et al., 2009; Tew et al., 2009; Marie and Trainor, 2014).

Further exploring more complex musical stimuli, He and Trainor (2009) utilized an oddball paradigm that was designed so that detection of the missing fundamental pitch would elicit an MMN. No MMN was elicited at 3 months of age, but the authors concluded that a major developmental shift in cortical pitch representation develops shortly thereafter (He and Trainor, 2009). An MMN was elicited in both 4- and 7-month-olds, suggesting they were able to integrate harmonics into a single pitch percept and thus perceive pitch similarly to adults (He and Trainor, 2009).

Infants were able to hold separate memory traces for polyphonic music, and displayed a high voice preference in polyphonic music from 3 months (Tew et al., 2009; Marie and Trainor, 2014). Marie and Trainor (2014) hypothesize this high voice superiority may be innately mediated at the auditory nerve level. Again the MMN-like response to this more complex polyphonic stimulus was reported to have a longer developmental trajectory than the MMN elicited by a single tone stimulus (Tew et al., 2009; Marie and Trainor, 2014).

Jing and Benasich (2006) explored the developmental trajectories of the MMN and other components of the AEP up to 2 years of age, and reported significant morphological changes of the AEP around 6 months. The P150, N250, P350, and N450 were all reliably elicited after this age and the MMN became robust and more adult-like (Jing and Benasich, 2006). From 7 months of age infants were found to have the capacity to entrain to a rhythm, however the cortical level at which this entrainment occurred was not able to be determined (Cirelli et al., 2016). Given that the deeper layers of the auditory cortex are immature in infants under 6 months, it is likely that the components of the AEP (including the MMN) have different neural generators to those of adults (He et al., 2009b). This theory was supported by Hamalainen et al. (2011) who concluded that at 6 months infants begin to utilize analogous cortical areas to adults to detect pitch change in their auditory environments, including the frontal and supratemporal cortices and anterior cingulate cortex (located in the limbic structures). After this age, the MMN component elicited by tone pairs had adult-like latency, distribution, and source locations. The positive deflection that followed this MMN was thought to resemble the adult P3a that is related to an attention-switching mechanism (Hamalainen et al., 2011). Despite the relatively rapid development of the AEP components, the perceived emotional valence of a piece of music may not significantly affect the AEP in the first 12 months of postnatal life (Schmidt et al., 2003).

Extending the knowledge of the cortically recorded AEP components, the frequency following response (FFR) component indicates that essential subcortical structures utilized for tracking pitch are functional from birth (Jeng et al., 2016a,b). Tracking the development of the FFR across time, Jeng et al. (2016b) reported improved subcortical tracking accuracy and pitch strength at 3 months, supporting the hypothesis that neural circuitry involved in processing musical stimuli is refined through development and passive exposure (Jeng et al., 2016b).

### Young Children Aged 2–6 Years

The fMRI data included in this age range extend knowledge about global cortical and subcortical hemodynamic responses to musical stimuli. Prabhakar et al. (2018) described increased bilateral hippocampi activation in sleeping toddlers, in response to a previously heard lullaby. This result indicates that music stimulates sub-cortical memory substrates in young children (Prabhakar et al., 2018). In slightly older children, activation of the bilateral temporal lobes, frontal lobes, fusiform, and cerebellum were reported in response to music (Overy et al., 2004; Guerrero Arenas et al., 2016). Wernicke's area and the cerebellum were involved in the processing of both tonal and atonal music indicating involvement of language and motor areas of the brain (Guerrero Arenas et al., 2016). Further, Overy et al. (2004) reported a small right lateralized region that displayed higher activation during melody discrimination compared to rhythm discrimination (Overy et al., 2004).

Fujioka et al. (2006) longitudinally tracked the development of cortical auditory-evoked fields (AEF) of children aged 4–6 years across a year. Maturation of the AEF was indicated by shorter peak latencies and decreased amplitudes across sessions (Fujioka et al., 2006). It was also noted that the violin tone, compared to the noise burst, consistently elicited a larger peak amplitude. This indicates that greater neural resources are required for the processing of more complex auditory stimuli (Fujioka et al., 2006). The EEG data included in this age group also track the ongoing maturation of the AEP. The MMN however, continues to be less robust than its adult counterpart and its maturational time-course may vary depending on different auditory features (Putkinen et al., 2013). No correlation was found between the MMN and informal music activities, which may indicate that development of the MMN is not exposure-dependent (Putkinen et al., 2013).

Implicit knowledge of musical syntax begins to emerge during these early childhood years (Jentschke et al., 2008, 2014; Corrigall and Trainor, 2014). The early right anterior negativity (ERAN) component of the AEP is elicited by violations of musical syntax, and is typically followed by the N5 which is thought to reflect the process of harmonic integration into a mental representation of Western tonality (Koelsch, 2009). Jentschke et al. (2014) reported that at 30 months of age, the ERAN-like response had a longer latency, smaller amplitude, and broader scalp distribution than that observed in adults (Jentschke et al., 2014). The N5 was not observed until after 4 years of age (Jentschke et al., 2008). Corrigall and Trainor (2014) also reported an immature ERAN at 4 years in response to harmonic stimuli but no significant response elicited by irregularities in a melody-only stimulus (Corrigall and Trainor, 2014). These results highlight that young children demonstrate implicit, but immature, cortical representations of Western harmonic key membership before explicit behavioral sensitivity is observed (Jentschke et al., 2008, 2014; Corrigall and Trainor, 2014).

### Children Aged 7–12 Years

The implicit ability to process music according to Western tonal syntax music rapidly develops during the older childhood period, and is explored in both EEG and fMRI data (Koelsch et al., 2003, 2005; Jentschke et al., 2008). Children in this age group displayed an immature ERAN to strong syntactic irregularities, or syntactic irregularity at a cadence point (Koelsch et al., 2003; Magne et al., 2006; Jentschke and Koelsch, 2009). However, an ERAN was not elicited following a weak syntactic error or a syntactic irregularity in the middle of a musical phrase (Koelsch et al., 2003; Magne et al., 2006; James et al., 2015). Further, the ERAN continued to have a long latency and different scalp distribution compared to its mature counterpart (Jentschke and Koelsch, 2009). Interestingly, at 9 years, male children displayed a left predominance for the ERAN and female children a more bilateral distribution, compared to adults' right predominance (Koelsch et al., 2003). This distribution is similar to the cortical distribution observed in the processing of some aspects of language in children at the same age (Koelsch et al., 2003). Thus there may be overlapping neural generators for music and language processing in children, and the electrical correlates of musical syntactic processing were reported to emerge earlier than those of language (Koelsch et al., 2003; Jentschke and Koelsch, 2009). A music/language overlap was further supported by James et al. (2015) who recorded a left dominant centro-posterior negativity, reminiscent of the semantic (language specific) mismatch N400 in response to an ambiguous musical syntactic error.

FMRI data demonstrated that this syntactic processing takes place in a network of frontal, fronto-temporal, and limbic structures, with a pattern of right hemisphere activation in 10-year-olds, similar to that observed in non-musically trained adults (Koelsch et al., 2005). Irregularities of musical syntax were hypothesized as having a role in generating emotions in music and stimulated a neural network that includes the pars opercularis (part of the inferior frontal gyrus) and the inferior venterolateral pre-motor cortex (located in the frontal lobe) (Koelsch et al., 2005). The included fMRI and EEG data seem to present contradictory results related to the localization of musical syntax processing. However study participants' different ages may indicate a maturational time course, or the different scanning methodologies may be impacted by temporal and spatial factors (Koelsch et al., 2003; Jentschke and Koelsch, 2009).

The majority of references included in this age group explore melodic and harmonic processing, but cortical immaturity also impacts the perception of tempo and rhythm. Parviainen et al. (2019) explored hemispheric processing of stimuli present at different inter-stimulus intervals in 7-8-year-old children. Similar to adults, tones presented monaurally elicited a stronger response in the contralateral auditory cortex. A right hemisphere preference was noted, potentially indicating immaturity of the left hemisphere at 8 years (Parviainen et al., 2019). The protracted development of rhythmic processing was further evident as stimuli presented at a fast pace (short inter-stimulus interval) did not elicit a distinct neuro-electrical response or an induced beta-band entrained oscillation in this age group (Fox et al., 2010; Cirelli et al., 2014). It is hypothesized that the absence of these evoked responses to stimuli presented with short inter-stimulus intervals may result from immaturity in both the auditory and motor cortex (Cirelli et al., 2014).

The AEP in this age cohort was predominated by the P1 and N2 peaks reflecting auditory sensory processes, with the N1 not recorded at 4 years but functional in a 9-year-old cohort to both slow and rapid stimulus presentation (Ceponiene et al., 2002). The N1 generators in the 9-year-olds were anterior to those observed in adults, and this relatively late maturation of the N1 may reflect the immaturity of the frontal lobes at this age and protracted development of the neuronal generators required for auditory sensitivity and orienting (Ceponiene et al., 2002).

### Adolescents Aged 13+ Years

The AEP continues to mature into late adolescence (Yamazaki et al., 2018). By late adolescence, right cortical hemispheric dominance for tonal stimuli resembles adult processing, as does the stability of the N1, P1, N2, and P2 components of the AEP components elicited by single tone stimuli (Shahin et al., 2010; Yamazaki et al., 2018). The presence of an audible movie soundtrack continued to have a degrading effect on the latency of the N1 and MMN peaks at 16-17 years of age, despite the stability of the amplitudes of the other AEP components (Mahajan and McArthur, 2011). The development of phase-locking to external auditory stimuli also strengthened with age, with phase-locking in the higher frequency bands (upper-beta and gamma) occurring later than lower bands. This aligns with the typical development of the power of spontaneous oscillation that shifts from lower to higher frequencies (Shahin et al., 2010).

## Discussion

It is evident that beyond being merely functional at birth, an infant's auditory system is sensitive to a variety of musical elements. After the immediate postnatal period, complex musical competence is acquired effortlessly throughout childhood but remains immature into adolescence (Trehub, 2003; Perani et al., 2010; Mahajan and McArthur, 2011; Jentschke et al., 2014). The following discussion explores considerations for clinical practice in music therapy with children who have sustained a severe ABI, specifically for early cognitive rehabilitation for children presenting with a disorder of consciousness (DOC).

The impact of nature vs. nurture has not been explored in this review. Indeed research on the specific effects of musical exposure and experience were excluded because it is increasingly acknowledged that formal music tuition/training in children results in neuroplastic changes observed in adulthood (See Appendix C) (Gaser and Schlaug, 2003; Putkinen et al., 2013). However, the presence of an AEP in infancy, in infants who were not reported to have had intentional pre-natal music exposure, may indicate an innate ability for musical processing (Cheour et al., 2002; Stefanics et al., 2009; Lordier et al., 2019). For example, the presence of the MMN component of the AEP to pitch deviants indicates an ability to recognize pitch change, the foundation of melody processing (Stefanics et al., 2009; Háden et al., 2015). However, the infant AEP is likely mediated by different neural pathways than its adult counterpart (Werner, 2012). Described grossly, in adults, musical stimuli project from the cochlear to the thalamus and then the auditory cortex, followed by triggering of cortical attentional resources toward musical events that may be of interest. Meaning may then be attributed to the music depending on previous experience and emotional context (Tervaniemi and Brattico, 2004). Despite the relative maturity of the auditory brainstem at birth, the cortex remains immature with the frontal lobes continuing to mature into adolescence (He and Trainor, 2009; Mahajan and McArthur, 2011; Werner, 2012). It is hypothesized that prior to maturation of the thalamo-cortical pathways after 6 months of age, AEPs likely stem from reticular formation inputs to the cortex (Werner, 2012). The reticular formation plays a fundamental role in arousal and consciousness (Mangold and Das, 2020). This may be of clinical relevance because music may support increasing arousal or potentially sustaining states of optimal arousal, through stimulating pre-attentive sensory processing.

The transmission of musically evoked response through the immature brain occurs more slowly and less efficiently than similar responses in adults (Shahin et al., 2010). The components of the infant AEP have a longer latency and variable amplitude (Cheour et al., 2002; He et al., 2007, 2009a). Increased myelination, maturation of synaptic connections, and synaptic pruning lead to increased localization and efficiency of musically elicited neuro-electrical transmissions (Magne et al., 2006; Fujioka and Ross, 2008; Giedd and Rapoport, 2010). ABI in children is also known to negatively impact the processing speed of cognitive tasks in children (Anderson et al., 2012). Children presenting with a DOC require stimulation to support optimal arousal, yet overstimulation may have a detrimental impact on recovery (Pool and Magee, 2016). The pacing and duration of musical experience should be closely monitored, particularly in children who may have a limited repertoire of behaviors to indicate distress or agitation. Repetition of musical material may support optimal processing. The impact of competing sensory stimuli needs to be considered carefully when working with children as pre-attentive processing of a single tone stimuli was reported to be less accurate when a competing auditory stimulus was present (Mahajan and McArthur, 2011).

Current evidence indicates that in healthy right-handed, non-musically trained adults, the prime musical architectures are predominantly right lateralized and are subserved by additional left hemisphere structures (Sihvonen et al., 2019). Language is somewhat the mirror of this with a left hemisphere dominance (Levman et al., 2017). Further, whilst some anatomical overlap is noted, the processing of music and speech is functionally separate in non-musically-trained adults (Abrams et al., 2011; Peretz et al., 2015). This distinction is less clear in children (see Figure 2) (Overy et al., 2004; Tervaniemi and Brattico, 2004; Putkinen et al., 2013; Jentschke et al., 2014; James et al., 2015; Jeng et al., 2016b; Sihvonen et al., 2019). While there is some evidence of a right lateralization in melodic processing, children may process music and language more similarly, and utilize a greater network of cortical structures, than adults (Koelsch et al., 2003; Perani et al., 2010; James et al., 2015). Koelsch et al. (2003) reported that at 9 years of age, males displayed a left predominance, and females a bilateral pattern, for the processing of musical syntax. It was concluded that there may be overlapping generators for the processing of musical and language syntax in children (Koelsch et al., 2003). This pattern of bilateral activation supports that a right hemispheric insult may not impair the ability to process music in children in the same way it would in adults. Further, the electrical responses elicited by musical-syntactic processing were recorded at a younger age than a comparative electrical response to language-syntactic processing (Koelsch et al., 2003; Jentschke et al., 2008). This supports a hypothesis that complex musical processing may mature earlier than language processing (Koelsch et al., 2003).

Retention of cognitive skills established before the brain insult is increasingly well-documented (Crowe et al., 2019) and it is feasible that the same retention of musical skills may occur. Gentle et al. (2015) reported the clinical case of a 5-year-old female who retained singing functioning following a profound ABI that resulted in an incomplete right hemispherectomy. The neurological resilience of song functioning was highlighted to support an argument for the potential of song in cognitive rehabilitation (Gentle et al., 2015). For the child described, it was likely that the foundational skills of musical processing were established prior to the age at insult.

The implicit understanding of melody and harmony based around Western scale structures (musical syntax) is thought to be one of the most sophisticated perceptive musical abilities, and subsequently develops later in childhood than the processing of basic musical elements (Magne et al., 2006; Jentschke and Koelsch, 2009; Trehub, 2013). Development of an implicit understanding of tonal syntax requires enculturation through exposure. Thus the discriminatory response (ERAN) elicited by syntactic incongruence does not begin to emerge until after 2 years of age (see Figure 2) (Jentschke et al., 2014). This development of a pre-attentive understanding of major-minor tonal music supports an argument for the use of melodically and harmonically regular music in the early cognitive rehabilitation for those children who have been pre-morbidly enculturated to Western tonal music. Such music may provide meaningful sensory stimulation whilst reducing the potential for overstimulation and associated cognitive fatigue. Nursery rhymes typically follow the rules of Western musical syntax, are simple, and may have emotional connections with memories of early parent-child musical interactions. Further, nursery rhymes are sung to support early cognitive, social, emotional, and motor development in infants and toddlers (Trainor, 2002). Thus, nursery rhymes may be an ideal stimulus to maximize arousal and awareness, to support optimal functional recovery in infants and children following ABI, especially in the presence of language deficits (Rosenfeld and Dun, 1999; Bower and Shoemark, 2012; Pool and Magee, 2016). Similarly, for adolescents, an argument could be made for the use of harmonically simple, pre-morbidly familiar popular music that follows the rules of Western tonal music.

Extending this evidence of enculturation and pre-attentive responses to musical syntax, emotional responses and increased arousal may result from suspension and fulfillment of musical expectancies (Koelsch, 2005; Koelsch et al., 2005; Steinbeis et al., 2006). An emerging ERAN before 3 years of age would indicate that children might also experience a similar arousal or emotional response. This has clinical implications as harmonic and melodic manipulation of pre-composed songs can be used to create expectancy and increased arousal (Bower, 2010; Bower et al., 2014). For example Bower et al. (2014) reported that a “recruitment” response in a 10-year-old child was elicited in quintessential moments in familiar songs in the early stages of recovery following profound ABI. This observed behavioral response indicated an increase in arousal and was interpreted as an intentional, cognitively mediated response from a child in an overwhelming state of low-responsiveness and agitation (Bower et al., 2014).

Studies included in this review indicate that similarly to adults, infants and children process music throughout a broad network of cortical and sub-cortical structures and that discriminatory responses to minor and dissonant music are recorded from infancy (Virtala et al., 2013). However, it is vital to note that simply being able to differentiate discrete musical elements does not equate with musical preference nor that there is any meaning attributed to this beyond that there is simply a change in the auditory stimuli (Trehub, 2013; Virtala et al., 2013). The majority of references included in this review employed “scientific reductionism” in defining music and explaining the neural structures and networks involved in its perception and processing (Tivadar and Murray, 2019, p. 85). Individual components of music (rhythm, pitch, harmony, etc.) are necessarily operationalized under experimental conditions (Hunt, 2015). While this deconstructed approach to exploring music for this review was fundamental in objectively analyzing brain responses, it potentially produces a limited definition of music as merely “organized sound” (Cross, 2003, p. 106). Further, the social or emotional meaning attached to musical experiences in children; from parental singing in infancy, to personal music listening as a leisure activity, to musical contexts for identity formation in adolescence was not captured in this review. Especially in a clinical scenario, music and musical experiences should be conceptualized as actions that occur within individual, social, and broader cultural contexts (Elliott and Silverman, 2013). Thorough considerations should be given to past musical experiences, familial music preferences, and the ecology of the clinical setting. There is also an argument for whole music (e.g., song or musical work) to be utilized in therapeutic interventions following ABI as the multiple combined elements that form music are likely to stimulate a broader range of intact cortical function than a single element alone to support increased arousal and awareness (Koelsch et al., 2005; Guerrero Arenas et al., 2016).

## Limitations of This Review and Narrative Synthesis

There are a number of limitations of the search strategy employed in this review. Firstly, the search strategy was kept intentionally broad to maximize the data included in the narrative. This has however resulted in a large number of references being included in the narrative and a subsequent reduction in the detail that could be explored. The use of the search term “EEG” rather than AEP may have reduced the number of references available for inclusion. The search terms were based around scanning technologies utilized within music therapy research, and not the elicited responses as may be the preferred search strategy in psychology and auditory sciences. Further the diverse scanning methods, ages, musical stimuli, and study designs included in the review mean it was not possible to undertake meta-analysis. Studies that research the processing of single and/or pure tone stimuli were included and it is acknowledged that some researchers and clinicians may not classify pure tone stimuli as music/musical. However, the authors argue that a single tone is the building block of melody and thus the decision was made to include these references. Additionally, in clinical practice in very early brain injury rehabilitation, single tones may be introduced to assess responsiveness to a basic musical stimuli (Magee et al., 2015).

EEG was the most utilized scanning methodology with 33/46 of the included references using this technology and this likely represents the ease of use and cost effectiveness of this method (Tivadar and Murray, 2019). It does however indicate a paucity of hemodynamic-based research (fNIRS and fMRI) that reports the neural substrates of music processing in infants and children. Further, low participant numbers were noted in a significant proportion of studies, particularly fMRI, fNIRS, and MEG. The cost, availability, and feasibility of having children remain still for extended periods of time likely explains these low participant numbers but again indicates the lack of hemodynamic-based research methodology in this area.

There is an uneven age distribution in the included references. The 0-2 years age group contained 24/46 of the included references and even within this group the majority of infants were <6 months of age. This likely reflects the pragmatic aspects of scanning methodology and ease of scanning young infants whilst asleep. Nonetheless, this age disparity represents large gaps in our understanding of musical processing in older infants, children, and adolescents. The role of gender in musical processing also warrants further exploration with only one reference exploring this (Koelsch et al., 2003). The total cerebral volume of female children peaks ~3 years earlier than males, and while correlation between volume and function is complex, it may be possible that musical syntax is not the only aspect of musical processing that is impacted by sex (Koelsch et al., 2003; Lenroot and Giedd, 2006).

## Clinical Implications of the Neural Processing of Music in Children

The included narrative and discussion highlights the following considerations for music therapists working with pediatric neurologic populations:

- Infants are born (at term) with the neural foundations for processing basic musical elements. However, we do not know what meaning infants place on these musical experiences. It is likely that meaning is developed through interactions with an attuned partner and through ongoing exposure (Trevarthen and Aitken, 2001; Malloch and Trevarthen, 2018).
- Infants have a high voice preference when presented with polyphonic music and considerations should be given to the use of accompanying instruments, for example guitar or piano accompaniment should be pitched lower than a vocal melody.
- The responses to more complex musical stimuli have a more protracted developmental trajectory and considerations should be given to this when working with infants and young children following ABI to reduce the potential for overstimulation. Acapella singing may be optimal to stimulate intact musical processing whilst supporting fundamental interpersonal interaction.
- Following ABI, pre-morbidly attained musical skills are likely to remain intact. Given this and the emerging evidence that some elements of music processing may develop before corresponding language processing, music may be an ideal therapeutic medium to support early arousal, awareness, and cognitive stimulation for pediatric patients with an ABI.
- It is reasonable to hypothesize that an ABI will impair the ongoing maturation of music processing, and thorough consideration must be given to the age and musical development time point at which a child sustained the ABI when designing music therapy programs.
- The use of syntactically simple, melodically and harmonically regular music, that does not violate musical syntax, may reduce the potential for cognitive fatigue and negative impacts of overstimulation in children and adolescents presenting with a DOC.
- Clinicians should consider the musical manipulation of syntax and its resolution to support increased arousal in children and young people presenting in states of low arousal (including DOC) following ABI.
- It is unlikely that an ABI sustained in a childhood will impact musical processing in the same way it would an adult, and therefore adult research is not immediately translatable to the pediatric population.

## Conclusion

A foundational understanding of the neurophysiological processing of music is essential for clinicians intending to utilize music as a therapeutic medium for infants, children, and adolescents following ABI. This knowledge encourages an understanding of what music processing skills may be present pre-morbidly, how these skills are impacted by the ABI, and finally how the ABI may alter the ongoing development of musical processing abilities. While evidence supporting the use of music interventions for adults with a DOC following ABI is expanding, the pediatric population remains severely under-represented in the current literature. The human brain reaches ~90% of adult size by 5 years but the relationships between the size of a particular brain area and its functional capacity is “staggeringly complex” as experience plays a substantial role in connectivity and functionality (Lenroot and Giedd, 2006, p. 726; Tau and Peterson, 2010). As such, key researchers promote vigilance against “adultomorphism” (Trehub, 2013, p. 1). Whilst there are some similarities in the way children and adults process music, results of this review add weight to the argument that adult knowledge is not immediately translatable to the pediatric population. Infants and children process music more slowly, use different cortical areas for this processing, and potentially lack the memory and emotional associations experienced by adults (Koelsch, 2005). Despite this, the capacity for processing the basic musical elements is present from birth and therefore may be argued to be innate. Evidence supports that rehabilitative interventions should commence in the early days and weeks following ABI to hasten emergence to consciousness and maximize functional recovery (Catroppa et al., 2016; Giacino et al., 2018). Results of this review support that music may be ideally situated to achieve this aim. It is noted that this review only explored the processing of music, and additional stimuli and/or rehabilitative therapies should be considered as part of multidisciplinary care of this vulnerable population.

There is an obvious need for further music neuroscience research with children and music therapy research with children following ABI. Research that longitudinally tracks the developmental trajectory of musical milestones would be enormously beneficial to the field. Research that maps the electrical processes and hemodynamic responses to whole music, as opposed to discrete musical elements, and explores potential emotional responses to music is also required. Exploring the processing of music in children following ABI and the potential disruption caused by an ABI to the developmental trajectory of music processing would develop clinical practice in music therapy. Finally, research that objectively describes any effect of music-based interventions targeted for consciousness rehabilitation with children following ABI is needed.

## Data Availability Statement

The original contributions presented in the study are included in the article/Supplementary Material, further inquiries can be directed to the corresponding author/s.

## Author Contributions

JB ran the database searches with assistance from a medical research librarian, undertook data extraction, and drafted the manuscript. JB, WM, CC, and FB screened titles and abstracts in the first round screening, undertook full text review in the second round screening, and collaboratively developed the narrative synthesis and considerations for clinical practice. WM, CC and FB edited the manuscript. All authors contributed to the article and approved the submitted version.

## Conflict of Interest

The authors declare that the research was conducted in the absence of any commercial or financial relationships that could be construed as a potential conflict of interest.

## Footnotes

^1^See Supplementary Material for a description the included scanning methods.
