# Supplementary material for: The Neurophysiological Processing of Music in Children: A Systematic Review With Narrative Synthesis and Considerations for Clinical Practice in Music Therapy
Source: Front Psychol. 2021 Apr 15;12:615209. doi: 10.3389/fpsyg.2021.615209 (PMC8081903; doi:10.3389/fpsyg.2021.615209)
Supplement: Supplementary file 1 [file Data_Sheet_1.pdf]

Supplementary Material – Included Imaging Techniques

Electroencephalography (EEG)

EEG was the first of the non-invasive methods for recording brain activity (Tivadar & Murray, 2019). EEG records spontaneous electrical activity that is largely generated by the postsynaptic potential of groups of pyramidal neurons (Kirschstein & Kohling, 2009; Rossini et al., 2019; Tivadar & Murray, 2019). EEG has excellent temporal resolution with electrical activity recorded at the scalp within milliseconds (Kujala & Näätänen, 2010).

EEG – Auditory evoked potential (AEP)

Whereas EEG is a recording of spontaneous neuro-electrical activity, an AEP (also called an auditory event related potential- ERP) is an electrical response that is time-locked to an external auditory stimulus and is extracted from the ongoing brain activity captured by EEG (Remijn, Hasuo, Fujihira, & Morimoto, 2014; Tivadar & Murray, 2019). An AEP is an objective measure of a cortical level response with excellent temporal accuracy (Risetti et al., 2013). A critique of the AEP in the cognitive neurosciences is the difficulty in accurately localizing the neural generators, given voltages recorded at the scalp typically reflects electrical activity from multiple brain areas (Kappenman & Luck, 2016; Remijn et al., 2014). In adults, an AEP consists of well-defined components (e.g. *N100, P100, N200, P200* etc) however there are substantial differences between the AEP composition in young children and adults, likely related to ongoing neuro-developmental changes (Ceponiene, Rinne, & Naatanen, 2002; Remijn et al., 2014).

EEG - Mismatch negativity (MMN)

An MMN is a component of the AEP that occurs when the incoming stimulus does not match the prediction (He, Hotson, & Trainor, 2009). When a deviant stimulus is presented within a string of sounds (the 'oddball' paradigm) the MMN is elicited approximately 150-200ms after the change onset (in adults). The MMN is considered to reflect early sensory memory processing involved in matching incoming stimuli with a previously processed stimuli, and reflects an automatic change detection process (He et al., 2009).

EEG – Early right anterior negativity (ERAN)

The ERAN is a component of the AEP that is elicited by irregularities in music-syntactic processing. Music that violates the complex rules governing Western major/minor tonal music elicits an ERAN. The presence of an ERAN indicates representation of musical syntax in the long-term memory. The ERAN typically has an anterior scalp distribution and right hemisphere weighting, and

is elicited approximately 150-250mns after the presentation of the syntactic violation (Koelsch, 2009).

EEG - Frequency following response (FFR)

The FFR is a scalp-recorded measurement that reflects the action potentials of the sub-cortical structures that are phase-locked to an auditory stimulus. The FFR is part of the auditory brainstem response (Jeng, Lin, Chou, et al., 2016; Jeng, Lin, & Wang, 2016).

Magnetoencephalography (MEG)

Electrical brain activity results in the generation of very small magnetic fields that are measured using superconducting sensors within MEG scanners (Fujioka, Ross, Kakigi, Pantev, & Trainor, 2006; Horwitz, Friston, & Taylor, 2000; Rossini et al., 2019). Similar to EEG, the source of the magnetic fields is the post synaptic potentials of cortical pyramidal neurons, and because MEG signals are obtained directly from neuronal electrical activity MEG, similarly to EEG, is a direct measure of neuronal activity (Proudfoot, Woolrich, Nobre, & Turner, 2014). MEG affords the same excellent temporal resolution as EEG, however higher spatial resolution and subsequent sources localization is recorded as the magnetic fields do not undergo the same distortive effects observed in EEG (Proudfoot et al., 2014).

MEG - Auditory evoked field (AEF)

The auditory evoked magnetic field (AEF) is the MEG counterpart to the AEP and provides useful information about when and where sound is neurologically processed (Fujioka et al., 2006).

Functional near infrared spectroscopy (fNIRS)

Near infrared spectroscopy (NIRS) is an optical brain imaging technique that records changes in the ratio of oxygenated and de-oxygenated hemoglobin. A neurophysiological response to a stimulus results in a change in oxygen consumption in the area of focal activity. Oxygenated and de-oxygenated hemoglobin have different absorption spectra in the near-infrared light range, which is recorded from the scalp at pre-set measurement points (Irani, Platek, Bunce, Ruocco, & Chute, 2007; Obrig & Villringer, 2003). NIRS has excellent temporal resolution however the spatial resolution is poor compared to more invasive and advanced neuro-imaging techniques such as fMRI. Functional NIRS (fNIRS) is utilized to image the functional physiology of cortical activation in response to an external stimulus (Obrig & Villringer, 2003). As a hemodynamic method, fNIRS is a secondary or surrogate measure of brain response.

Functional magnetic resonance imaging (fMRI)

Magnetic resonance imaging (MRI) is based on the magnetization properties of protons within water molecules in organs, tissue and bones. It uses strong magnetic fields to generate structural and anatomical images (Logothetis, 2008). Functional MRI (fMRI) is utilized to study brain activity in response to an external stimulus and is arguably the "best tool we have for gaining insights into brain function" (Logothetis, 2008, p. 877). Focal areas of neural activation have an increased or decreased requirement for oxygen, and the protein hemoglobin transports oxygen to or from the required area of activation. Oxygenated and de-oxygenated hemoglobin have different magnetic properties that can be detected during fMRI (Dale, Brown, Semelka, & Brown, 2015; Horwitz et al., 2000). This blood oxygenation level dependant contrast (BOLD) is the most commonly measured signal in fMRI (Horwitz et al., 2000). The primary strength of fMRI is the relatively high spatial resolution as both cortical and subcortical networks can be recorded. However, because it is a measure of a surrogate brain response temporal resolution is reduced compared to EEG and MEG (Logothetis, 2008).

References

Ceponiene, R., Rinne, T., & Naatanen, R. (2002). Maturation of cortical sound processing as indexed by event-related potentials. *Clinical Neurophysiology, 113*(6), 870-882. doi:http://dx.doi.org/10.1016/S1388-2457%2802%2900078-0

Dale, B. M., Brown, M. A., Semelka, R. C., & Brown, M. A. (2015). *MRI : basic principles and applications* (Fifth edition. ed.): Wiley Blackwell.

Fujioka, T., Ross, B., Kakigi, R., Pantev, C., & Trainor, L. J. (2006). One year of musical training affects development of auditory cortical-evoked fields in young children. *Brain, 129*(10), 2593-2608. doi:http://dx.doi.org/10.1093/brain/awl247

He, C., Hotson, L., & Trainor, L. J. (2009). Development of infant mismatch responses to auditory pattern changes between 2 and 4 months old. *European Journal of Neuroscience, 29*(4), 861-867. doi:https://dx.doi.org/10.1111/j.1460-9568.2009.06625.x

Horwitz, B., Friston, K. J., & Taylor, J. G. (2000). Neural modeling and functional brain imaging: An overview. *Neural Networks, 13*(8-9), 829-846. doi:10.1016/S0893-6080(00)00062-9

Irani, F., Platek, S. M., Bunce, S., Ruocco, A. C., & Chute, D. (2007). Functional Near Infrared Spectroscopy (fNIRS): An Emerging Neuroimaging Technology with Important Applications for the Study of Brain Disorders. *The Clinical neuropsychologist, 21*(1), 9-37.

Jeng, F. C., Lin, C. D., Chou, M. S., Hollister, G. R., Sabol, J. T., Mayhugh, G. N., . . . Wang, C. Y. (2016). Development of subcortical pitch representation in three-month-old chinese infants. *Perceptual & Motor Skills, 122*(1), 123-135. doi:https://dx.doi.org/10.1177/0031512516631054

Jeng, F. C., Lin, C. D., & Wang, T. C. (2016). Subcortical neural representation to Mandarin pitch contours in American and Chinese newborns. *The Journal of the Acoustical Society of America, 139*(6), EL190. doi:http://dx.doi.org/10.1121/1.4953998

Kappenman, E. S., & Luck, S. J. (2016). Best practices for event-related potential research in clinical populations. *Biological Psychiatry: Cognitive Neuroscience and Neuroimaging, 1*(2), 110-115. doi:10.1016/j.bpsc.2015.11.007

Kirschstein, T., & Kohling, R. (2009). What is the source of the EEG? *Clinical EEG and neuroscience, 40*(3), 146-149. doi:10.1177/155005940904000305

Koelsch, S. (2009). Music-syntactic processing and auditory memory: Similarities and differences between ERAN and MMN. *Psychophysiology, 46*(1), 179-190. doi:0.1111/j.1469-8986.2008.00752

Kujala, T., & Näätänen, R. (2010). The adaptive brain: A neurophysiological perspective. *Progress in Neurobiology, 91*(1), 55-67. doi:10.1016/j.pneurobio.2010.01.006

Logothetis, N. K. (2008). What we can do and what we cannot do with fMRI. *Nature, 453*(7197), 869-878. doi:10.1038/nature06976

Obrig, H., & Villringer, A. (2003). Beyond the visible-imaging the human brain with light. *Journal Cerebral Blood Flow and Metabolism, 23*(1), 1-18. doi:10.1097/01.WCB.0000043472.45775.29

Proudfoot, M., Woolrich, M. W., Nobre, A. C., & Turner, M. R. (2014). Magnetoencephalography. *Practical Neurology, 14*(5), 336. doi:10.1136/practneurol-2013-000768

Remijn, G. B., Hasuo, E., Fujihira, H., & Morimoto, S. (2014). An introduction to the measurement of auditory event-related potentials (ERPs). *Acoustical Science and Technology, 35*(5), 229-242. doi:10.1250/ast.35.229

Risetti, M., Formisano, R., Toppi, J., Quitadamo, L. R., Bianchi, L., Astolfi, L., . . . Mattia, D. (2013). On ERPs detection in disorders of consciousness rehabilitation. *Frontiers in Human Neuroscience, 7*(775). doi:10.3389/fnhum.2013.00775

Rossini, P. M., Di Iorio, R., Bentivoglio, M., Bertini, G., Ferreri, F., Gerloff, C., . . . Hallett, M. (2019). Methods for analysis of brain connectivity: An IFCN-sponsored review. *Clinical Neurophysiology, 130*(10), 1833-1858. doi:https://doi.org/10.1016/j.clinph.2019.06.006

Tivadar, R. I., & Murray, M. M. (2019). A primer on electroencephalography and event-related potentials for organizational neuroscience. *Organizational Research Methods, 22*, 69-94. doi:10.1177/1094428118804657
