# Supplementary material for: The Neurophysiological Processing of Music in Children: A Systematic Review With Narrative Synthesis and Considerations for Clinical Practice in Music Therapy
Source: Front Psychol. 2021 Apr 15;12:615209. doi: 10.3389/fpsyg.2021.615209 (PMC8081903; doi:10.3389/fpsyg.2021.615209)
Supplement: Supplementary file 2 [file Data_Sheet_2.docx]

Appendix A. Medline Search Strategy

| **#** | **Searches** |
| --- | --- |
| 1 | exp *Brain/ah, ph |
| 2 | exp *Cerebrum/ah, ph |
| 3 | exp Psychophysiology/ |
| 4 | 1 or 2 or 3 |
| 5 | *Music/ |
| 6 | Acoustic Stimulation/ |
| 7 | exp Pitch Perception/ |
| 8 | (sing or singing or sung or song*1 or sang).tw,kf. |
| 9 | (music* or rhythmic* or melod* or harmon*).tw,kf. |
| 10 | 5 or 6 or 7 or 8 or 9 |
| 11 | exp Magnetic Resonance Imaging/ |
| 12 | exp neuroimaging/ or functional neuroimaging/ or brain mapping/ |
| 13 | exp diagnostic techniques, neurological/ or electroencephalography/ |
| 14 | 11 or 12 or 13 |
| 15 | (newborn* or new-born* or baby or babies or neonat* or neo-nat* or infan* or toddler* or pre-schooler* or preschooler* or kinder* or boy or boys or girl or girls or child or children or childhood or pediatric* or paediatric*).af. |
| 16 | 4 and 10 and 14 and 15 |
| 17 | exp neurodevelopmental disorders/ or autistic disorders/ |
| 18 | Cochlear Implantation/ |
| 19 | exp hearing disorders/ or deafness/ |
| 20 | exp neural prostheses/ or cochlear implants/ |
| 21 | exp animals/ not human*.sh. |
| 22 | (rat or rats).af. |
| 23 | 16 not (17 or 18 or 19 or 20 or 21 or 22) |
| 24 | limit 23 to (english language and yr="1999 - Current") |

Appendix B. Inclusion & Exclusion Criteria

| Inclusions criteria | Exclusion criteria |
| --- | --- |
| - Studies reported in peer reviewed journals | - Review/meta analysis articles - Articles reported in non peer reviewed forums - Conference abstracts - Theoretical and/or discussion papers |
| - Studies reporting brain imaging data about the processing of music in healthy children   - Includes MRI, fMRI, fNIRS, EEG, MEG | - Studies reporting behavioral responses to music where there is no inclusion of imaging data |
| - Child participant/s aged 0-18 years (inclusive) | - Adult participants aged >18 years - Preterm/premature infants aged <37 weeks gestation - Animal studies |
| - Neurologically healthy, neuro-typical participants | - Participants with a neurodevelopmental, neurodegenerative and/or neurological disorder, including (but not limited to); - Autism/Autism Spectrum Disorders - Retts Syndrome - Fetal alcohol syndrome - Agenesis of the corpus callosum - Cerebral palsy - Attention Deficit Hyperactivity Disorder (ADHD) - Dyslexia - Epilepsy - Schizophrenia or other mental health diagnosis - Specific language impairment/ developmental language disorder - Participants with a peri-natally acquired brain injury - Participants with an acquired brain injury, including those presenting with reduced consciousness/disorder of consciousness |
|  | - Participants with hearing loss, including participants with a cochlear implant |
| - Studies reporting the neural processing of music/musical stimuli, or where the described intention of the authors is to report the neural processing of music/musical stimuli | - Studies reporting language perception or processing - Studies reporting non musical rhythms, including EEG rhythms, cardiac/ECG rhythms |
| - Music/musical stimuli:   - Discrete components of music (melody, rhythm, harmony etc)   - Whole musical experiences (as opposed to discrete musical components)   - Auditory stimuli described in musical terms, for example prosodic elements of speech, prosodic elements of mother-infant interactions, melodic stimuli, rhythmic stimuli   - Single tones | - Auditory stimulus that would not be classified as music/musical within the clinical music therapy context, for example;   - Noise/auditory noise   - Sound (not defined in musical terms)   - Beeps   - Speech stimuli |
| - Studies that report the emotional, social, cultural effects or experiences of music/musical stimuli |  |
| - Studies reporting passive music perception/exposure/listening | - Studies reporting active music participation including playing a musical instrument, singing and/or dancing - Studies reporting the impact of any music education, musical expertise, learning a musical instrument, vocal training and/or singing lessons, and/or targeted music exposure/interventions on brain substrates, cognitive processes or neural processing - Studies where the participants are required to be an active participant in a musical experience, including finger tapping, dancing |
| - Studies reported/written in English language | - Studies where the full-text article is not available in English language |
| - Articles published within the last 20 years | - Articles published before (and including) 1998 |

APPENDIX C. Rationale for Exclusion Criteria

| **Exclusion Criteria** | **Reason for exclusion** |
| --- | --- |
| Adults aged ≥18 years | Children will not have had the same experience dependant neuroplastic changes as adults relating to music listening. Children’s brains are undergoing a period of rapid cortical development compared to adults and brain imaging results may not be comparable (Wilke et al., 2003). Studies that include adults AND children will be included and the data separated at the data extraction stage of the review process. |
| Premature infants <37 weeks gestation | Pre-term birth is known to results in altered maturation of both white and grey brain matter, and further result in reduced cortical volume |
| Participants with a neurodevelopmental, neurodegenerative and/or neurological disorders, including (but not limited to);   - Autism / Autism Spectrum Disorder - Retts Syndrome - Fetal alcohol syndrome - Agenesis of the corpus callosum - Cerebral palsy - Attention Deficit Hyperactivity Disorder (ADHD) - Dyslexia - Epilepsy | Participants with a diagnosed neuro-developmental disorder may have alternative/adapted/disrupted musical substrates compared with neuro-typical peers. |
| Participants with peri-natally acquired brain injuries | Participants with peri-natally acquired brain injuries may have neuro-cognitive deficits and alternative/adapted/disrupted musical substrates compared with neuro-typical peers. |
| Participants with severe to profound hearing loss | Participants with a diagnosed hearing loss may have an impaired ability to hear and process auditory stimulation |
| Participants with a cochlear implant | Children born profoundly deaf who have received either a single or double cochlear implant may have non-typical auditory processing |
| Animal studies | There is insufficient data exploring complex sound/music processing in animal brains and comparing this to complex sound/music processing in the developing brains of human infants/children/adolescents |
| Studies reporting neural responses to ‘noise’ | In a clinical setting, noise is not defined as music/or musical |
| Studies exploring the impact of music education, musical expertise or intentional/controlled music exposure | It is increasingly acknowledged that formal music tuition/training in children results in neuroplastic changes observed in adulthood (Gaser & Schlaug, 2003; Putkinen et al., 2013). |
| Language perception or processing that does not include descriptions about musical components | In adults studies, shared and distinct neural substrates for the processing of music and language have been found (Peretz & Zatorre, 2005), however, this review is focused only on the neural processing of music as distinct from language. |
